# PhenoWell®—A novel screening system for soil‐grown plants

**DOI:** 10.1002/pei3.10098

**Published:** 2023-02-09

**Authors:** Ji Li, Michael A. C. Mintgen, Sam D'Haeyer, Anne Helfer, Hilde Nelissen, Dirk Inzé, Stijn Dhondt

**Affiliations:** ^1^ Department of Plant Biotechnology and Bioinformatics Ghent University Ghent Belgium; ^2^ Center for Plant Systems Biology VIB Ghent Belgium; ^3^ Discovery Sciences VIB Ghent Belgium; ^4^ Screening Core VIB Ghent Belgium

**Keywords:** abiotic stress, Arabidopsis, growth and development, high‐throughput screening assays, maize, nutrients, phytohormones, plant leaves, seedlings, soil

## Abstract

As agricultural production is reaching its limits regarding outputs and land use, the need to further improve crop yield is greater than ever. The limited translatability from in vitro lab results into more natural growth conditions in soil remains problematic. Although considerable progress has been made in developing soil‐growth assays to tackle this bottleneck, the majority of these assays use pots or whole trays, making them not only space‐ and resource‐intensive, but also hampering the individual treatment of plants. Therefore, we developed a flexible and compact screening system named PhenoWell® in which individual seedlings are grown in wells filled with soil allowing single‐plant treatments. The system makes use of an automated image‐analysis pipeline that extracts multiple growth parameters from individual seedlings over time, including projected rosette area, relative growth rate, compactness, and stockiness. Macronutrient, hormone, salt, osmotic, and drought stress treatments were tested in the PhenoWell® system. The system is also optimized for maize with results that are consistent with Arabidopsis while different in amplitude. We conclude that the PhenoWell® system enables a high‐throughput, precise, and uniform application of a small amount of solution to individually soil‐grown plants, which increases the replicability and reduces variability and compound usage.

## Graphical abstract

The PhenoWell® system allows screening for the effect on soil‐grown plants of compounds added via irrigation and a wide range of environmental conditions, like drought stress and different soil types.

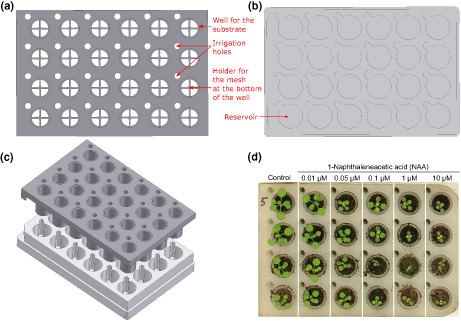

## 1 INTRODUCTION

In nature, plants are in permanent interaction with their environment and, because of their sessile nature, must constantly adapt to changes in their surroundings by adjusting their growth and development. Among the multitude of factors influencing plant growth, a large part is related to the substrate the plants grow in. For land plants, soil is the natural substrate which consists of different combinations of properties including water and nutrient availability, texture, and compaction for distinct soil‐grown plants (Brady et al., 2010). In addition, soil is a complex microbial ecosystem that is important for plant growth (Hardoim et al., 2008), stress resistance (De Zelicourt et al., 2013), and immunity (Van Wees et al., 2008).

Plant phenotyping entails the high‐throughput extraction of multiple growth parameters from considerably large plant populations in a short time. In Arabidopsis, the traits obtained from top‐view images such as projected rosette area (PRA), rosette compactness, and rosette stockiness have been used to evaluate plant growth over time (Vanhaeren et al., 2015). The PRA is the top‐view rosette area, which reflects the total rosette size of an Arabidopsis plant. The PRA has been shown to correlate with plant responses to various stresses such as salt, osmotic, drought and oxidative stress in in vitro and in‐soil studies (Dhondt et al., 2014; Clauw et al., 2015). The compactness, or surface coverage, refers to the ratio between the PRA and convex hull area (Jansen et al., 2009), while the stockiness represents the roundness of the rosette (Jansen et al., 2009). Together, compactness and stockiness reflect the architecture and shape of the rosette. When an image‐based plant phenotyping is integrated in a liquid‐handling platform, plant growth in response to chemical or stress treatments can also be assessed in a high‐throughput fashion. Whereas automated phenotyping of plants grown in soil has been well established, little work has been done to test the effect of chemicals when added to water or the nutrient solution of these plants (Dalal et al., 2019; Granier et al., 2006; Lee et al., 2018; Skirycz et al., 2011; Verbraeken et al., 2021). The large volumes of the pots or trays in which plants are grown not only require relatively large amounts of often expensive chemical compounds but also limit the throughput.

Considering these limitations of working with plants grown in soil, until now, most studies to test the effect of chemicals on plant growth were performed by adding them to in vitro growth medium. One successful in vitro screen for Arabidopsis growth regulators performed in multi‐well plates identified 587 growth‐inhibiting and 102 growth‐promoting compounds from a library containing 10,000 compounds (Rodriguez‐Furlán et al., 2016). The activity of 16 compounds was confirmed on a series of commercial crops like tomato, lettuce, and maize, but all growth experiments, including those in crops, were performed under in vitro conditions (Rodriguez‐Furlán et al., 2016). Another study evaluated the uptake of different trehalose‐6‐phosphate (T6P) analogs via the roots in Arabidopsis using an in vitro hydroponic system, which was translated into a spraying application on wheat plants grown in soil, for which a higher grain yield and drought tolerance was observed (Griffiths et al., 2016). Those results illustrate the potential to translate findings from in vitro conditions into soil‐grown plants. However, in vitro experiments, especially those using high‐throughput screening, generated many positive results that were not confirmed in soil‐grown plants (Köhl et al., 2020) or risked filtering out results that depended on an interaction with the environment, especially soil characteristics (Rouphael et al., 2018), making follow‐up validation more time and labor consuming. To a large extent, the inability to translate findings from in vitro into soil was due to substrate properties. Additionally, most in vitro experiments were performed with sealed Petri dishes to prevent contamination. Consequently, the plants grow in a saturated air space, and gas exchange with the surrounding was restricted, causing CO_2_ deprivation in the sealed Petri dishes. Not surprisingly, significant transcriptional and physiological changes in many pathways, including photosynthesis, respiration, and starch accumulation, were observed in plants in vitro‐grown in sealed Petri dishes as compared with their counterparts grown in aerated cultures (Banerjee et al., 2019). In addition, the medium and Petri dishes are transparent and expose the root system to light, which alters the plant development (Novák et al., 2015; Xu et al., 2013). These results underlined the need for new and easy‐to‐handle growth systems using natural growth conditions.

The PhenoWell® system presented here was designed to perform high‐throughput phenotyping experiments with small seedlings grown in soil. The aim of the system development was to combine natural plant growth conditions with a high flexibility on individual seedling treatments, without losing the advantages of a high‐throughput screening approach. In combination with a dedicated image analysis pipeline, the system enabled the determination of multiple growth parameters from individual soil‐grown plants, visualizing the plant response to specific treatments over time. We successfully tested various liquid treatments of Arabidopsis seedlings by applying different phytohormones, salinity, and osmotic stress. Furthermore, we analyzed the effect of different soil types, nutrient availabilities, and the alteration of the watering regime on plants grown in the PhenoWell® system.

The PhenoWell® platform was also adapted for soil‐grown maize seedlings. Maize is the second most important cereal in providing feed and food. Although cereals share with Arabidopsis several conserved molecular networks regulating growth (Nelissen et al., 2016), differences in wiring and activity of such networks are mediating the large morphological differences between Arabidopsis and monocots (Conklin et al., 2019). Maize leaves have emerged as model organs to understand organ growth in monocots due to their large growth zone with a linear organization (Nelissen et al., 2016). Chemical components, such as phytohormones, are normally applied to maize grown in soil by foliar spraying (Huang et al., 2019) or hydroponic/soil application in a relatively large quantity (Ren et al., 2022; Shi et al., 2019), which were either inaccurate or inefficient, especially for molecules of which only small amounts were available. Here, we showed that the PhenoWell® system is also suited to study the effect of molecules and growth conditions on soil‐grown maize seedlings.

## 2 MATERIALS AND METHODS

### 2.1 Plant material and growth conditions

For all Arabidopsis experiments, *Arabidopsis thaliana* (L.) Heyhn. accession Columbia‐0 (Col‐0) seeds were used. Before sowing, the seeds were stratified in water for 2 days at 4°C to break dormancy and achieve uniform germination. For all maize experiments, maize inbred line B104 seeds were used. Before sowing, the maize seeds were primed with water in 50‐ml falcon tubes at 25.5°C for 18 h. The primed seeds were germinated in wetted filter paper rolls at 25.5°C in the dark until most of the grown‐out primary roots reached around 1 cm. Equally germinated seeds were selected for the follow‐up experiments.

The experiments were performed at 21°C (Arabidopsis) or 25.5°C (maize) in a climate‐controlled growth chamber with a 16‐h light and 8‐h dark cycle and a light flux of 100 μmol m^−2^ s^−1^ (Arabidopsis) or 170 mmol m^−2^ s^−1^ (maize). In the room, a relative humidity of 50% (Arabidopsis) or 55% (maize) was maintained throughout the experiments.

### 2.2 PhenoWell® plate preparation, soil type selection, and seed sowing

**FIGURE 1:**
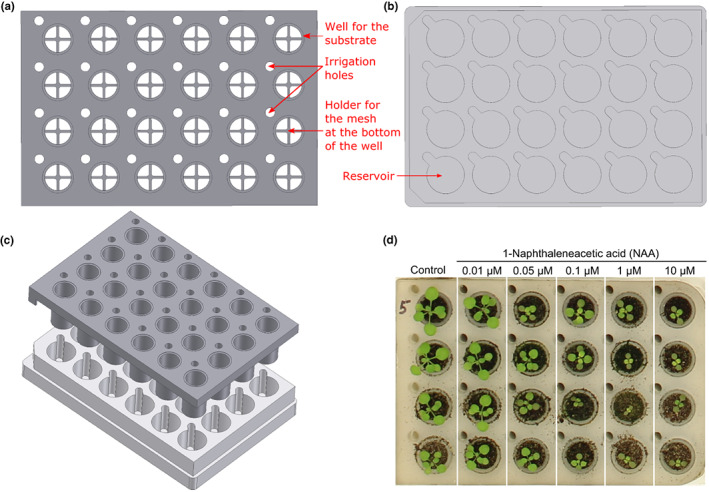
Design of the PhenoWell® plates. (a) Technical drawing of the insert plate of the PhenoWell® system. (b) Drawing of the corresponding adapter plate. (c) 3D rendering of the PhenoWell® design showing the assembly of the insert and adapter plate. (d) Example of a treatment of seedlings with five concentrations of the artificial auxin 1‐Naphthaleneacetic acid.

The PhenoWell® system consists of an insert plate (adapted from PELCO Prep‐Eze) that contains the seedlings and is made from polypropylene (Figure 1a), and a custom‐made adapter plate that allows liquid manipulation and is made from polyoxymethylene (Figure 1b). A plastic mesh with a pore size of 420 μm is placed at the bottom of each well of the insert plate to keep the substrate in place. The PhenoWell® insert plates of Arabidopsis have 24 independent wells. The dimensions of the Arabidopsis PhenoWell® plate is 12.7 cm × 8.5 cm (length x width) and the volume of each well is 2.2 cm^3^. The PhenoWell® insert plates of maize have six independent wells. The dimensions of the original maize PhenoWell® plate is the same as the Arabidopsis counterpart while the volume of each well is 11.5 cm^3^. The depth of the well of the modified maize PhenoWell® plate is increased by 1.5 times, which resulted in a well volume of 17.25 cm^3^, while the dimensions of the optimized maize PhenoWell® plate is the same as the original design.

The wells of the insert plate were filled with sieved potting soil (N.V. Van Israel, Geraardsbergen, Belgium) mixed with sand in a ratio of 3:1 (v/v). Sand was added to the soil to achieve fast water absorption. The nutrient levels of four soil types (potting soil, poor soil, propagation soil, and enriched soil) used for testing soil‐type effects on growth are listed in Table S1. The effect of different soils on plant growth were tested in the PhenoWell® system. For the soil nutrient measurement, NO_3_^−^ and NH_4_^+^ were extracted with water and measured via ion‐chromatography (European Committee for Standardization, 2001). P and K were analyzed using an ammonium acetate extraction method in combination with an inductively coupled plasma optical emission spectrometry (ICP‐OES) measurement (European Committee for Standardization, 2001). Seedlings grown in poor soil and propagation soil showed a reduced PRA compared with those grown in potting soil (Figure S1a). In addition, all plants grown in poor soil also showed clearly visible stress symptoms on the leaves and petioles (Figure S1b). Growth in enriched soil resulted in the largest plants with an increased PRA compared with the plants grown in potting soil (Figure S1a,b). The potting soil contained optimal nutrients to maintain growth for the whole duration of the experiment (14 days) and was therefore also used in most of the other experiments and served as the control, except for the phosphate experiment. The soil for the phosphate experiment was the low phosphate soil from N.V. De Ceuster Meststoffen (DCM), Belgium. To assure equal filling of the wells, the substrate was first evenly distributed over the whole plate and the excess was removed with a straight edge. Then the soil was compressed by applying a light pressure on the substrate in the individual wells using a custom‐made device with pins, each with the diameter of one well. Thereby, a homogeneous compactness was achieved in all wells of the plate. This procedure was repeated until all wells were equally filled. The initial soil water humidity (SWH, in g water/g dry soil), which is also called gravimetric soil water content, was calculated based on the weight of soil water divided by the dry weight of the same soil sample. The soil sample was taken during the filling of the plates.

To sow the Arabidopsis plants, one seed was slightly pushed in the soil in each well of the insert plate. To sow the maize plants, a hole in the soil of each well was made to fit the equally germinated seeds with 1 cm root, which were subsequently covered with the surrounding soil. To obtain efficient germination, 1.25 ml (Arabidopsis) or 5.5 ml (maize) of water was added to each reservoir of the adapter plate (Figure 1b). The insert plate with the soil was then placed in the adapter plate (Figure 1c) and both parts were taped together with watertight plastic tape to prevent excessive water evaporation from the border wells of the plate and to avoid growth differences between plants in border and center wells. Then, the weight of the filled plate was measured to enable easy calculation of the daily SWH. To ensure optimal germination conditions, the plates were placed in the growth chamber and covered with transparent plastic boxes for 3 days, after which the covers were removed and the plants continued to grow under normal atmospheric conditions.

### 2.3 Irrigation and treatment of the PhenoWell® plates

The water in the reservoirs in the adapter plate was replenished daily through the holes located next to the wells in the insert plate until the desired soil water content was achieved. For Arabidopsis, the target soil water content was 0.95 g water/g dry soil, while for maize, the target was 1.5 g water/g dry soil (1.05 g water/g dry soil for the modified maize PhenoWell® system). The water was then taken up quickly via capillary forces by the soil to reach the root system of the seedling. No leaching or residual water in the receiver could be found after watering. Because of the small volume of soil present in the wells, daily watering was required to prevent excessive drying of the soil substrate during the experiment. The required volume of water was calculated daily for each plate individually, taking into account the current weight of the plate and the initial SWH and weight of the filled plate after sowing. For this, a repetition pipet was used and the volumes were rounded to 100 μl based on the accuracy of the pipette. Assuming equal filling with soil and a comparable water evaporation from the individual wells within one PhenoWell® plate, each well received the same amount of water.

For treatment of Arabidopsis seedlings at 5 and 7 days after sowing (DAS), the plants were not watered but 0.8 ml of phytohormone solution was added to the reservoir of every well. For treatment of maize seedlings, 2 ml of chemical solution was added when the first leaf had appeared and 2 days after appearance. The day after the treatments, no water was applied to the plates. For each treatment, five different concentrations can be investigated next to the control for Arabidopsis experiments (Figure 1d). At least four plates were used for each Arabidopsis experiment with four plants grown in one column receiving the same concentration of the compound and were treated as a replicate. To avoid a possible position effect, the column of each treatment was moved one column to the right on consecutive plates (red and blue rectangles in Figure S2). This experimental setup resulted in 96 plants per experiment, containing six columns of four plants per applied concentration. The solutions for the phytohormone, osmotic stress, salinity, and nitrogen fertilizer treatments, including the control (water) solutions, were prepared with 0.1% DMSO to improve the uptake via the roots and to increase the solubility of the compound. For the phosphate experiment, each well received 1.25 ml of KH₂PO₄ solutions once at the start of the experiment (0 DAS). The KH₂PO₄ solutions for the phosphate experiment were prepared with water supplied with 50 mg·L^−1^ NH_4_NO_3_ to ensure sufficient nitrogen supplement. All the KH₂PO₄ solutions with different concentrations including the 0 mg·L^−1^ KH₂PO₄ control had the same concentration of potassium (supplemented by KCl) as the 64 mg·L^−1^ KH₂PO₄ solution. The concentrations of the solutions for Arabidopsis experiments are listed in the Table S2.

### 2.4 Drought stress experiment

An SWH of 0.95 g water/g dry soil for Arabidopsis and of 1.05 g water/g dry soil for maize was maintained during the establishment of the seedlings. For Arabidopsis, the daily irrigation regime was changed to 0.75, 0.50, or 0.35 g water/g dry soil from five DAS (the day when the leaf appears) onward and kept at these levels till the end of the experiment. For maize, drought was applied from the third day after placing the germinated seeds in soil, by watering the plants every other day to reach an SWH level of 0.75, 0.55, or 0.35 g water/g dry soil.

### 2.5 Image acquisition, processing, maize measurement, and data analysis

**FIGURE 2:**
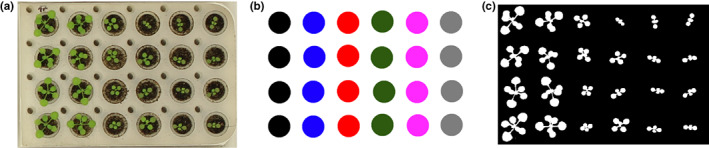
Overview of the image‐analysis pipeline. (a) An example of an RGB image, cropped to a single PhenoWell® plate, taken during the experiment. (b) Mask file in which dots with the same color indicate the position of wells with the same treatment. (c) Binary image, generated after image segmentation, which is used to measure the different growth traits.

Throughout the experiments, the PhenoWell® plates were kept at the same position below the camera system, which allowed the automated analysis of the RGB images over time to extract the growth parameters for individual seedlings (Figure 2a). Canon EOS 50D SLR cameras, mounted 130 cm above the plates between the light tubes in the top of the growth cabinet, were used and pictures were taken every 24 h at noon. The field of view of one camera covered an area containing 20 PhenoWell® plates. The images were analyzed using an in‐house developed image‐analysis algorithm, which extracted the PRA, perimeter, convex hull area to calculate the rosette parameters, including rosette compactness and rosette stockiness, throughout the experiment for individual Arabidopsis plants. The script of this algorithm and the calculation of the rosette parameters were described in our previous paper (Dhondt et al., 2014). First, a mask file, in which dots with the same color indicating the position of wells with the same treatment–genotype combination, was created to link each well to the corresponding metadata (Figure 2b). Next, plants were segmented from the background, resulting in a binary image in which plant pixels were represented in white and the background in black (Figure 2c). This image was then used to extract the different growth parameters: PRA, compactness and stockiness. Image pre‐processing and segmentation were performed with C++ scripts using the OpenCV image‐analysis library (www.opencv.org). Parsing of quantitative measurements of Arabidopsis experiments and further data analysis were performed using Perl scripts (www.perl.org). Graphs of the calculated data were automatically plotted using the graphing utility gnuplot (www.gnuplot.info).

For maize, the lengths of the first two leaves (immediately after appearance to when fully grown) were measured daily with a ruler. The leaf elongation rate was leaf length difference between two continuous time points divided by the time interval. The final plant fresh weight was measured by weighing the total plant with soil washed off on the last day the second leaf (leaf 2) length was measured.

### 2.6 Statistical analysis

Time series data were analyzed as repeated measurements using the residual maximum likelihood (REML) approach as implemented in Genstat v22. Briefly, a linear mixed model of the following form (random terms underlined) was fitted to the data: y = u + phenowell + treatment + time + treatment. time + phenowell.column + plant. time. The response variable y represents the repeated PRA, stockiness and compactness measurements. The blocking factors phenowell and phenowell.column as blocking factors represent the experimental design of the trials. The random term phenowell.column accounts for the pseudo‐replication. Even though we might have a total of 8 or 16 observations per treatment, these have a structure, and the treatment was randomized to the two or four columns and not to the single plants, making the columns serving as replicates. The factors treatment and time and in particular the treatment–time interaction term were of major interest here because we wanted to assess the differences between the treatments across the time series. The term plant. time represents the residual error term with dependent errors because the repeated measurements were taken serially in the same individual, causing correlations among observations. There were several variance–covariance structures that could be used for repeated measurements. The autoregressive correlation model of order 1 (AR1) was finally selected as best fitting model based on the Akaike's information criterion coefficient. The AR variance–covariance model assumed that correlation between observations decayed as the measurements were collected further apart in time. The first order referred to the fact that only recent (t‐1) observations affected the current observations made at timepoint t. Times of measurement were set as equally spaced, and the option of unequal variances across time was chosen when it improved the model fit. The significance of the fixed terms in the model and the significance of changes in difference between treatment effects over time were assessed using an approximate F‐test as implemented in Genstat v22.

End point data were analyzed by fitting a linear mixed model of the following form (random terms underlined): y = u + phenowell + treatment + phenowell.column + error. The significance of the fixed terms was assessed using an approximate *F*‐test as implemented in Genstat v22, followed by multiple comparison testing based on the Fisher's Protected Least Significant Difference, as implemented in Genstat v22.

## 3 RESULTS

### 3.1 Design of the PhenoWell® system

The PhenoWell® system represents a novel design for high‐throughput screening of the effect of environmental stress and chemicals applied in the soil on the shoot growth of seedlings. The following sections describe the use of the system for Arabidopsis; the platform was also successfully implemented for maize seedlings in the last two sections (PhenoWell® system optimized for maize seedlings and Modified design of the PhenoWell® system for drought treatments in maize).

The system consists of two complementary parts: an insert plate and a corresponding adapter plate (Figure 1a,b). When the insert plate is positioned inside the adapter plate, liquid can be taken up from the receivers in the adapter plate to the wells in the insert plate by soil capillarity (Figure 1b,c). Next to the wells, the insert plate contains additional holes to allow the addition of water (and/or dissolved chemicals) to the reservoirs of the adapter plate underneath. Through this design, each well has a separate reservoir, which makes it possible to apply different treatments to individual plants grown in the same PhenoWell® plate. The PhenoWell® system offers a high flexibility regarding the timing and frequency of the treatment, as chemicals can be given without interfering with the germination and the early development of the seedlings. In addition, the complete device, with insert and adapter plate, has the dimensions of a common multi‐well plate, making it compatible with standard liquid handling systems.

### 3.2 Optimization of the treatment regime

In order to determine the optimal treatment regime not interfering with the establishment of the young seedlings, while offering a long observation period, two different treatment setups were tested in a pilot experiment. A 10‐μM solution of the synthetic auxin 1‐naphthaleneacetic acid (NAA) was applied to the plants at five and seven DAS (early treatment) or at seven and ten DAS (late treatment).

**FIGURE 3:**
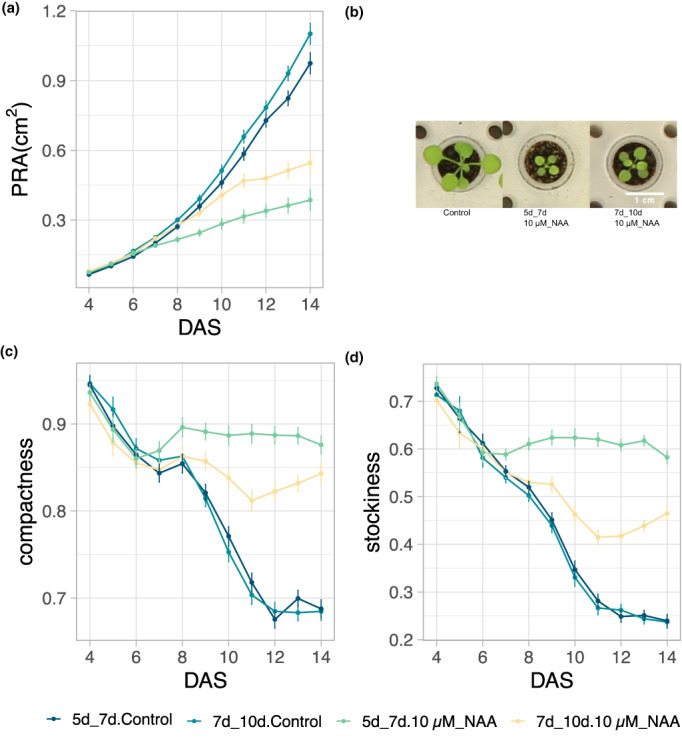
Determination of the optimal treatment regime for the PhenoWell® assay. (a) Changes in PRA of the early (5 & 7 DAS) and the later treatment (7 & 10 DAS) with 10 μM NAA over time. (b) Representative pictures at 14 DAS of control plants (left), a plant treated with 10 μM NAA at 5 & 7 DAS (middle) and at 7 & 10 DAS (right). (c, d), Means of compactness (c) and stockiness (d) measurements over time. Error bars indicate standard error of the mean (*n* = 4). DAS: Days after sowing; 5d_7d: Treatment at 5 and 7 DAS; 7d_10d: Treatment at 7 and 10 DAS.

For both regimes, comparable trends were observed, showing a decrease in the PRA compared with the control treatment, but with the later treatment, plants responded about 3 days later than the early treatment (Figure 3). The early treatment did not only show earlier effects, but also resulted in a larger difference in the PRA at the end of the measurements at 14 DAS, with a decrease of 63% for the early treatment and 49% for the later treatment (Figure 3a,b). The PRA change over time of the early treatment was significantly different from that of the late treatment (repeated measurements analysis followed by *F*‐test (RMA), *p* < .01). For the parameters compactness and stockiness (Figure 3d,e), the effect of the early treatment was significantly more pronounced than that of the later treatment (RMA, *p* < .01). Based on these results, it was decided to treat the plants at five and seven DAS for future experiments.

### 3.3 Phytohormones in the PhenoWell® system

**FIGURE 4:**
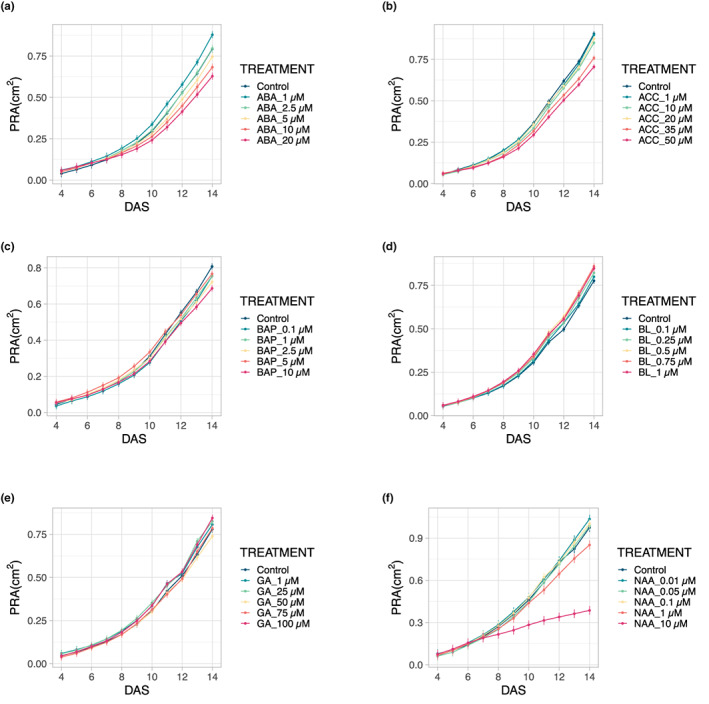
Changes in projected rosette area (PRA) after the treatment with different phytohormones compared with the control treatment in the PhenoWell® system. (a) Abscisic acid (ABA). (b) 1‐Aminocyclo‐propane‐1‐carboxylic acid (ACC). (c) 6‐Benzylaminopurine (BAP). (d) Brassinolide (BL). (e) Gibberellic acid 3 (GA). (f) 1‐Naphthaleneacetic acid (NAA). Error bars indicate standard error of the mean (*n* = 4).

Next, we analyzed the response of the seedlings to different phytohormone treatments in the PhenoWell® system. We applied abscisic acid (ABA), 1‐aminocyclopropane‐1‐carboxylic acid (ACC), 6‐benzylaminopurine (BAP), brassinolide (BL), gibberellic acid 3 (GA), and 1‐naphthaleneacetic acid (NAA) at five and seven DAS in various concentrations and compared the PRA to that of control plants. For all concentrations of ABA above 1 μM, a growth inhibitory effect was visible, which became more pronounced with increasing ABA concentrations and was significant for the 10‐μM and 20‐μM concentrations (RMA, *p* < .01, decrease of 15.7% and 23.1%, respectively) (Figure 4a). For all treatments with ACC, the precursor of ethylene, a growth reduction was found, which was also significant for the two highest concentrations (RMA, *p* < .01) with a decrease of 17.2% for the 35‐μM and 25.4% for the 50‐μM treatment (Figure 4b). Treatment of the seedlings with the synthetic cytokinin BAP resulted in a reduced PRA, which was significant for the application of 2.5, 5, and 10 μM BAP (Figure 4c, RMA, *p* < .01). Treatment with BL, a representative of the brassinosteroids, tended to have a growth promoting effect, but even the 1‐μM application was not significantly different from the control treatment (RMA, *p* = .094) (Figure 4d). For GA, a growth‐promoting trend was also observed for all concentrations, but most of the effects were not significant except for that of 25 μM GA (*p* = .014) (Figure 4e). The application of NAA resulted in no significant PRA change for the treatments below 10 μM; for the 10‐μM applications, a significant decrease in PRA (RMA, *p* < .01) of 63% was found (Figure 4f). In conclusion, application to soil‐grown seedlings of BL and GA, although not significant, increased the PRA, whereas treatments with all other tested hormones had a negative, dose‐dependent effect on plant growth.

### 3.4 The effect of different nutrient applications on growth performance

**FIGURE 5:**
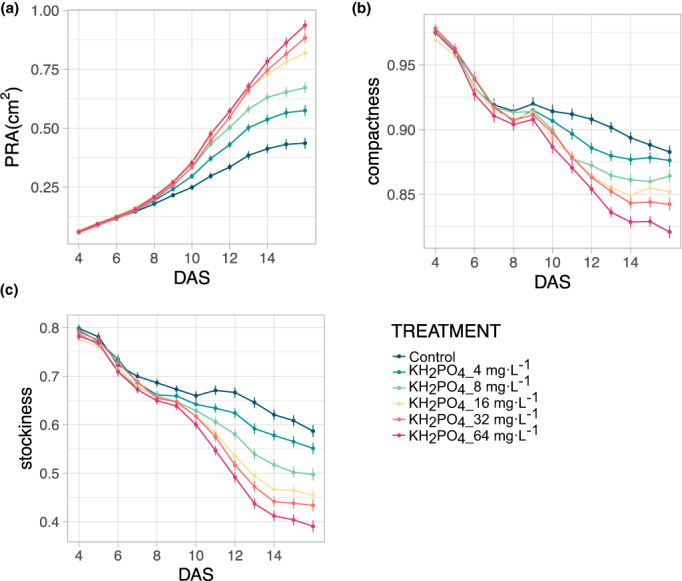
Effect of different phosphate concentrations on Arabidopsis seedling growth. (a–c), Changes in PRA (a), stockiness (b) and compactness (c) of plants exposed to different concentrations of KH_2_PO_4_ compared with control plants. Error bars indicate standard error of the mean (*n* = 4).

To determine if the seedlings also respond to the application of additional nutrients during growth, an experiment with different nitrogen fertilizers was first performed by applying KNO_3_, NH_4_Cl, and urea (CH_4_N_2_O) to plants grown in propagation soil. Compared with the water control treatment, none of the applied fertilizer treatments resulted in significant growth promotion, and at the highest concentration, even a strong reduction in the PRA was observed for all three fertilizer treatments (Figure S3). These results suggested that the nitrogen concentration was not limiting in the propagation soil during the time course of the experiment. We then tested the effect of different phosphate (KH₂PO₄) concentrations added to low phosphate soil on plant growth at 16 DAS which was longer than the normal experimental duration because of slower plant growth under low phosphate. Compared with control plants with no phosphate supplement, Arabidopsis plants with 4, 8, 16, 32, 64 mg·L^−1^ of KH₂PO₄ showed a significantly increased PRA (RMA, *p* < .01) by 31.2%, 54.4%, 80.7%, 100.8%, and 115.0%, respectively (Figure 5d). Similarly, significantly decreased rosette stockiness was observed in plants under 8, 16, 32, 64 mg·L^−1^ of KH₂PO₄ treatment by −15.4%, −20.5%, −25.8%, and −33.6% (Figure 5e), respectively, compared with control plants. The rosette compactness, which increased by 7.1% in 64 mg·L^−1^ KH₂PO₄ treated plants compared with control plants, also showed a clear dosage effect. In addition, the PRA, and rosette compactness and stockiness of KH₂PO₄ treated plants were statistically different from control plants from the lowest concentration (Figure 5f). This clear phosphate dosage‐dependent rosette growth response showed the capability and sensitivity of the PhenoWell® system to study plant growth under different phosphate treatments.

To sum up, we can conclude that the PhenoWell® system is well suited to study plant growth on soil with different nutrient contents.

### 3.5 Osmotic stress in the PhenoWell® system

**FIGURE 6:**
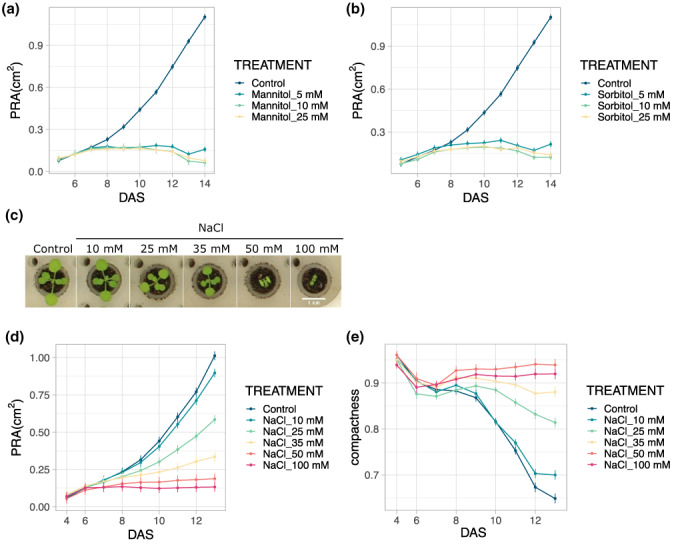
Effect of different osmotica and NaCl on plant growth in the PhenoWell® assay. (a, b), Changes in PRA of plants exposed to different concentrations of mannitol (a) and sorbitol (b) compared to control plants treated with water. (c) Representative pictures of plants treated with different NaCl concentrations. (d, e), Changes in PRA (d) and compactness (e) of plants exposed to different concentrations of NaCl. Error bars indicate standard error of the mean; (*n* = 4).

Osmotic stress caused by mannitol or sorbitol is often used in in vitro experiments as a proxy for water deficit in soil conditions. These osmotica reduce the osmotic potential of the growth medium and impair the water uptake of the roots. Here, we tested the effect of mannitol and sorbitol treatments in the PhenoWell® system. The addition of 5, 10, or 25 mM of mannitol or sorbitol resulted in a decrease of 0.01, 0.02, or 0.06 MPa, respectively, of the osmotic potential. The results showed a concentration‐dependent response to the applied molecules and the treatment with 5 mM of mannitol or sorbitol was sufficient to lead to a significant decrease in the PRA compared with the control treatment (RMA, *p* < .01) (Figure 6a, b).

### 3.6 Salinity treatment

Another way to apply osmotic stress to plants in vitro is the addition of salt to the growth medium, but here the toxicity of the sodium ions also contributes to the response. To study how seedlings respond to different salinity levels in the PhenoWell® system, NaCl was applied to the plants in five different concentrations. The results showed a clear concentration‐dependent effect on the growth and development of the seedlings (Figure 6c). The application of 10 mM NaCl was sufficient to reduce the PRA (albeit not significantly, RMA, *p* = .057) compared with the control plants, and for all higher concentrations, significant growth reductions were observed (RMA, *p* < .01) (Figure 6d). For the 25‐mM treatment, a PRA reduction of 42.8% was observed (Figure 6d). Additionally, larger compactness values were observed for higher NaCl concentrations (Figure 6e).

### 3.7 Drought stress

Next, we analyzed the suitability of the PhenoWell® system for drought stress studies. To this end, the daily watering regime was altered by decreasing the target SWH. Because the required amount of water is calculated based on the weight of the plate, one complete plate was used per applied SWH level. During the establishment of the seedling, a SWH of 0.95 g water/g dry soil was maintained, but from five DAS onward, the irrigation regime was changed to 0.75, 0.50, or 0.35 g water/g dry soil.

**FIGURE 7:**
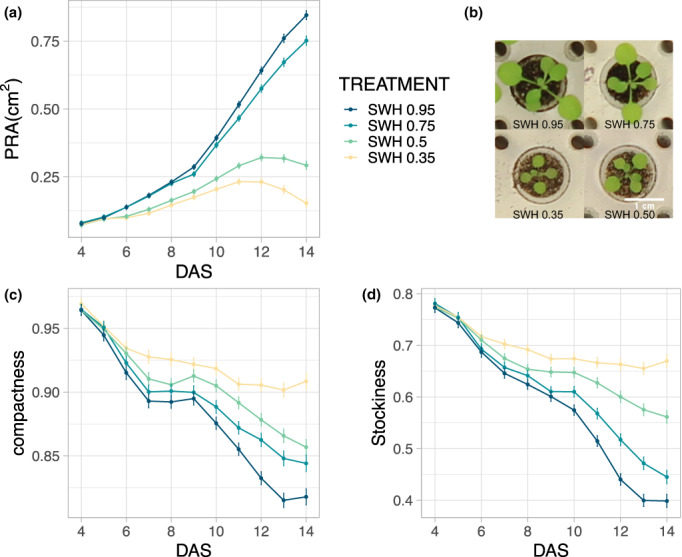
Effect of different drought stress regimes on plant growth. (a) PRA over time for different soil water humidity (SWH) levels. (b) Representative pictures of plants grown with different SWH levels. (c) Compactness for different SWH levels over time. (d) Stockiness for the different SWH levels over time. Error bars indicate standard error of the mean (*n* = 4).

The measurement of the PRA showed a clear dependency on the availability of water (Figure 7). The first differences were observed at eight DAS, only 3 days after the watering regime was changed (Figure 7a). At 14 DAS, the differences became clearly visible (Figure 7b) and the plants grown under the 0.75‐, 0.50‐, and 0.35‐SWH watering regime were 18.5%, 49.8%, and 59.4%, respectively, smaller compared with the control plants with 0.95‐SWH watering regime (Figure 7c). The treatments with the lower SWH levels of 0.50 and 0.35 g water/g dry soil additionally showed a flattening of the growth curve, whereas the 0.75‐SWH treatment still resulted in an exponential growth pattern (Figure 7a). Additionally, the compactness remained higher with decreasing SWH levels, which was an indication of smaller, bulkier plants with shorter petioles (Figure 7d). For all other tested SWH levels, decreasing the daily amount of applied water resulted in a significantly smaller PRA compared with the 0.95‐SWH control plants at 14 DAS. These results showed that the PhenoWell® system is suitable to perform drought stress experiments in soil, ranging from mild to severe drought.

### 3.8 PhenoWell® system optimized for maize seedlings

The PhenoWell® system was also developed for soil‐grown maize, due to its economical and scientific importance. The design of the maize PhenoWell® system is similar to that of the Arabidopsis counterpart except that the insert plate contains six wells with a volume of 11.5 cm^3^ each. To select homogeneous maize seedlings, maize seeds were primed in water and pre‐germinated in paper rolls. For the irrigation procedure, the target SWH was set to 1.5 g water/g dry soil to meet the relatively large water demand for maize seedlings. For treatments, chemicals were dissolved in 0.1% DMSO to target concentrations. In each well, 2 ml of solution was applied to the corresponding treatment groups at 0 day and 2 days after appearance of the first leaf (equal to 2 days before and the day of leaf 2 appearance, respectively). For the controls, the same amount of 0.1% DMSO without dissolved compound was applied. During the experiment, the length of leaf 2 was monitored to quantify the treatment effect on growth and by daily measuring leaf length. The maize PhenoWell® plates were randomized daily to avoid environmental differences and position effect.

**FIGURE 8:**
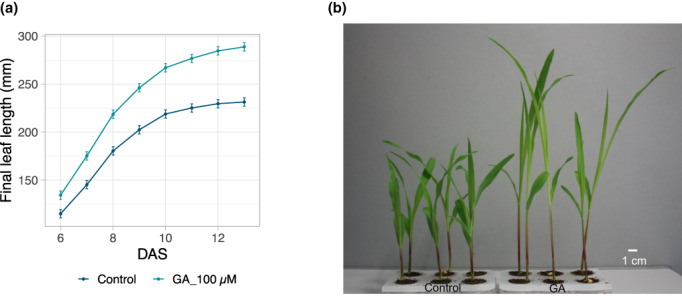
Leaf 2 growth of the maize B104 line with GA or control treatments in the maize PhenoWell® system. (a) Changes in leaf 2 length over time of B104 plants with 100 μM GA or control treatment. (b) The representative picture of B104 plants grown with control (left) or 100 μM GA (right) treatment. Error bars indicate standard error of the mean (*n* = 2).

The maize inbred line B104 was grown in the maize PhenoWell® system with treatments of different phytohormones such as ABA, ACC, NAA. Most of the phytohormones tested showed a reduction in leaf growth similar to, but less pronounced than, the observations in Arabidopsis (Figure S4), except for GA (Figure 8). In the PhenoWell® system, B104 maize seedlings were treated with 100 μM GA and compared with control plants. GA treatment led to a significantly (RMA, *p* < .01) enhanced length of leaf 2 from the first day after its appearance to the end of its growth (Figure 8a). Consequently, maize plants treated with GA were much larger compared with control plants (Figure 8b). These results show the applicability of the PhenoWell® system to investigate maize growth in response to exogenous treatments.

### 3.9 Modified design of the PhenoWell® system for drought treatments in maize

To optimize the PhenoWell® system for drought‐related experiments with maize seedlings, we increased the depth of the wells to 35 mm (volume of 17.25 cm^3^), which is 1.5 times larger than the original design. This enabled better root growth of the maize seedlings and improved the water availability. The modified maize PhenoWell® system was used to test leaf growth of maize seedlings under different SWH (1.05, 0.75, 0.55, 0.35 g water/g dry soil), set from five DAS onward, mimicking different drought conditions. A significant decrease in leaf 2 length for plants grown under 0.55‐SWH (RMA, *p* < .05) and 0.35‐SWH (RMA, *p* < .01) was observed compared with that under well‐watered conditions (Figure S5a). In addition, the fresh weight of plants under the 0.55‐ and 0.35‐SWH watering regime showed a significant 19.9% and 29.2% decrease, respectively, compared with well‐watered control plants (Figure S5b). These results showed that the modified PhenoWell® system is well suited to perform drought stress experiments in maize.

## 4 DISCUSSION

We demonstrated that the PhenoWell® system is a robust and relatively high‐throughput phenotyping system suitable to follow the growth of Arabidopsis and maize seedlings grown in soil. The multi‐well based system allows the extraction of multiple quantitative growth parameters over time from individual plants either treated with a chemical or a nutrient, or exposed to different environmental conditions. The PhenoWell® system can be seen as an intermediate step between in vitro experiments and subsequent experiments in larger pots with soil or in the field. Current soil‐phenotyping systems either use trays or small pots, which makes them less suitable to apply accurate concentrations of stress agents or chemical compounds (Dobrescu et al., 2017; Minervini et al., 2017). The space requirements for pots are usually high and relatively large quantities of chemical compounds are needed to reach a certain concentration in the pots, while trays can be smaller but do not allow for individual treatment of the plants growing in the same tray. In the current setup with 24 plants per PhenoWell® plate, more than 2200 Arabidopsis plants can be grown per m^2^. As such, the system easily can be installed in conventional climate‐controlled growth rooms. By daily imaging of the Arabidopsis seedlings and application of automated image analysis scripts, it is possible to measure various growth parameters, such as PRA, compactness and stockiness from individual plants over time. The PhenoWell® system is also compatible with the use of more advanced imaging devices, for example hyperspectral cameras, which allow the determination of the chemical composition of leaves, including concentrations of micro‐ and macronutrients or the leaf water content (Pandey et al., 2017). Additionally, the PhenoWell® system offers a high flexibility regarding timing and frequency of the treatments. In the experiment investigating the optimal treatment regime, we found that by treating the plants early, they have more time to respond to the treatment, which maximizes the observation period. As a result, subtle differences between individual treatments can be detected, without affecting the germination process. The experiment investigating the effect of different soil types and phosphate concentrations on plant growth indicated that the PhenoWell® system is suitable to test a wide array of natural growth substrates and their impact on plant performance.

As a proof of concept, we tested the application of NaCl, mannitol or sorbitol solutions in the reservoirs of the adapter plate, showing reproducible and dose‐dependent growth retardation for the different stress‐inducing treatments. Mannitol and sorbitol have been frequently used for in vitro experiments mimicking osmotic stress and our results show that both osmotica also work when applied to soil.

Next to treatments with osmotica, we showed that the PhenoWell® system is also well suited to perform more realistic drought stress experiments, by reducing or withholding irrigation in both Arabidopsis and maize. Because of the small size of the wells, the Arabidopsis plants in the drought stress experiments responded very fast to the limited water availability. In drought assays using bigger pots, the plants have shown a slower response, because it takes longer for the soil to dry and to reach the lower SWH level (Skirycz et al., 2011). In such larger pots, Arabidopsis plants exposed to a mild drought stress have shown a difference to the well‐watered control on the level of the individual leaf area between four and 5 days after changing the watering regime, whereas in the PhenoWell® system, a difference on the level of the entire rosette was observed within 3 days. We also found a clear difference between mild and severe drought conditions in the Arabidopsis assay. At 0.75‐SWH, a mild drought stress response was observed with a 20% reduction of PRA at 14 DAS, whereas the plants exposed to a SWH of 0.5 or 0.35 g water/g dry soil showed a non‐exponential growth pattern and a flattening of the growth curve at later time points. For maize, a significant difference in leaf growth and biomass was also found between well‐watered and severe drought‐treated maize. However, when exposed to mild drought stress, no significant difference in leaf growth was found during the time span of the assay. Overall, our work illustrates the suitability of the PhenoWell® system for drought tolerance screens.

Apart from these abiotic stress conditions, a series of phytohormones, which have been known to influence plant growth, was tested. For most of the applied compounds, a growth‐inhibiting effect was observed, which was not as drastic as reported for in vitro experiments. For example, Vaseva et al. (2018) have found that seedlings growing in vitro on 50 μM ACC have shown a decrease in rosette area of around 60% after 14 days, whereas in the PhenoWell® system, only a reduction of 25.4% was observed when the plants were treated twice with this concentration. In another study, Arabidopsis plants transferred to medium containing ABA have shown strong signs of senescence at concentrations of 5 μM, 14 days after transfer (Liu et al., 2016). In the PhenoWell® system, senescence was observed from concentrations of 20 μM ABA upward. A minor growth‐promoting effect was observed after the application of GA and BL to the plants. GA treatment slightly increased the leaf growth of Arabidopsis seedlings in the PhenoWell® system. This is consistent with other research where Arabidopsis plants with a foliar‐sprayed 50‐μM GA solution have shown a slight increase in rosette size (Ribeiro et al., 2012). Exogenously applied GA has shown more pronounced effects on germination (Ribeiro et al., 2012), stem elongation (Xu et al., 1997), and flowering time (Wilson et al., 1992). It has previously been shown that brassinosteroids positively affect growth by promoting cell division and cell expansion in the leaf (Nakaya et al., 2002; Zhiponova et al., 2013). Zhiponova et al. (2013) also have found that BL balances cell division and cell expansion during growth by modulating the progression of the cell cycle arrest front, the transition zone between cell division and cell expansion. The discrepancies between in vitro experiments and treatments using PhenoWell® can be explained by a rapid degradation of the compound in the substrate or by the adsorption to soil particles before it reaches the root system of the plant (Thompson & Goyne, 2012). Soil is already a whole biological system by itself, which is populated by various microorganisms that influence plant development (Bishnoi, 2015), and this microbiome can modify or degrade applied molecules (Asemoloye et al., 2017; Mandelbaum et al., 2008). Furthermore, the distribution of the phytohormone in soil may affect the accessibility of the phytohormone by roots. This may explain the relatively high concentrations of BL required to observe effects on the phenotypic level.

We also optimized the Phenowell® system for maize seedlings. In the maize PhenoWell® system, we tested phytohormones like ABA, NAA, and ACC on B104 maize seedlings for which similar growth responses were observed compared with Arabidopsis. However, in contrast to the effect on Arabidopsis, GA application in the PhenoWell® system drastically stimulated the growth of maize seedlings. The reason why maize seedlings grown in soil were more sensitive to GA application than Arabidopsis plants remains unknown.

As the Phenowell® system works with soil as a growth substrate, some drawbacks of currently used in vitro assays are overcome. Most importantly, plants are grown more close to natural conditions and in a non‐saturated air environment (Xu et al., 2013). Furthermore, the roots are protected from light. Compared with experiments in pots and trays, the irrigation process is more accurate, because each plant is watered individually, whereas in classical pot experiments, the plants have been usually watered per tray containing multiple pots. All these aspects result in more controlled growth conditions and facilitate the translation of results obtained from the PhenoWell® system into experiments in bigger pots or agronomical applications.

The relatively high‐throughput PhenoWell® system can be used for numerous applications based on the in soil behavior of seedlings. An example is the growth screening of CRISPR mutant collections or RILs in response to different soils, treatments, or chemicals. Another promising application of the system is the confirmation in soil conditions of initial hits from a high‐throughput chemical or biostimulant screen performed in vitro. Basic research with small molecules often relies on the synthesis of these compounds, which makes them expensive and hinders experiments in bigger pots. Because the wells in the PhenoWell® plate only have a volume of 2.2 cm^3^ for Arabidopsis and 11.5 cm^3^ for maize, smaller quantities of the chemicals are needed. Another common method to treat plants with chemical or biological molecules is the spraying of the molecule on the leaves (Preininger et al., 2018). With this application method, it is, however, difficult to predict the exact amount of the compound the plant is exposed to, as this depends on developmental parameters like the uptake through the leaf's cuticle or the shoot surface area (Fernández & Brown, 2013). Another problem is the treatment of individual plants with different chemicals and concentrations using a spraying application, since it is difficult to ensure that the compound is only applied to the designated plant, without affecting surrounding seedlings. Additionally, when the molecule remains on the leaf surface after the spraying application, exposure to light can affect its stability. By applying the compound in the substrate through irrigation, the compound is protected from light and all plants in the PhenoWell® plate are exposed to the same quantity of the chemical or stress agent. This is independent of the developmental stage of the plant because the concentration in soil is equal in all wells of the plate. Although, as mentioned above, adding the compound by watering also has some potential drawbacks, we believe that in an academic setting, this application method is preferred above spraying. In our standard growth conditions, the proposed 24‐well assay supports the growth and analysis of individual Arabidopsis seedlings, without overlap, up to around 14 DAS. However, the duration of the experiments can be easily extended up to 21 days when using a 6‐ or 12‐well plate set‐up. Furthermore, we also demonstrated that larger 6‐well plates can be used to grow and treat small seedlings of maize up to 16 DAS. However, one of the limitations of this system is that the limited well volume is on the one hand easy for chemical application but, on the other hand, not enough for the large maize root system. We minimized this limitation by measuring at a very early seedling stage until leaf 2 stops growing. The results we got from maize leaf 2 grown in the PhenoWell® system are similar to our previous results on maize leaf 4 grown in big pots (Sun et al., 2017).

In conclusion, the PhenoWell® system represents a reliable and compact soil‐phenotyping system that enables the determination of multiple growth parameters from individual seedlings over the time course of the experiment. The required amount of treatment solution is reduced to a minimum, while maintaining highly controlled growth conditions in a natural environment. Next to the liquid treatment with compounds added to the irrigation water, the assay can also be used to perform phenotyping experiments with a wide range of different environmental conditions, like drought stress and different soil types. The experiments performed in this study represent only a small fraction of the possible applications of the assay, because it may also be suitable to apply for example biostimulants, plant growth‐promoting rhizobacteria, herbicides, or heavy metals to seedlings. In conclusion, the PhenoWell® system can facilitate more extensive studies on the effect of growth‐promoting compounds on soil‐grown plants and may aid the translation of findings from in vitro studies into practical applications in soil.

## FUNDING INFORMATION

This work was funded by the “Bijzonder Onderzoeksfonds Methusalem Project” (BOF08/01 M00408) of Ghent University, a Postdoctoral Fellowship for S.D. by the Research Foundation–Flanders (FWO), and a PhD scholarship for Ji Li by the China Scholarship Council (CSC).

## CONFLICT OF INTEREST

A patent application (WO/2018/033603) on the described technology has been filed by the authors' institution.

## Supporting information


Table S1.
Table S2.
Figure S1.
Figure S2.
Figure S3.
Figure S4.
Figure S5.

## Data Availability

All data supporting the findings reported in this study are available in the paper.
