# Supplementary material for: PhenoWell®—A novel screening system for soil‐grown plants
Source: Plant Environ Interact. 2023 Feb 9;4(2):55–69. doi: 10.1002/pei3.10098 (PMC10243540; doi:10.1002/pei3.10098)
Supplement: Supplementary file 1 — Table S1. Table S2. Figure S1. Figure S2. Figure S3. Figure S4. Figure S5. [file PEI3-4-55-s001.pdf]

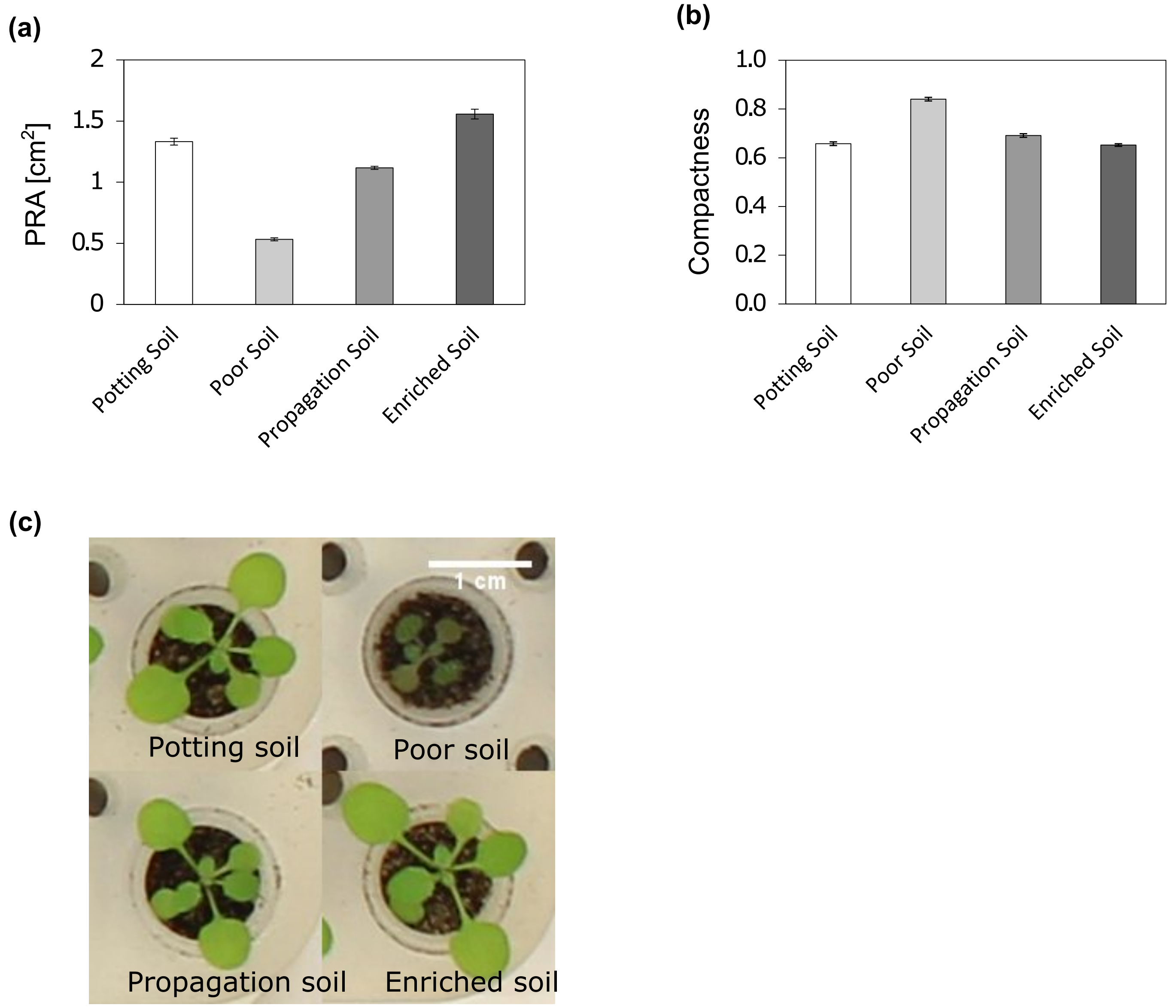

Figure S1. Effect of different soil types on Arabidopsis seedling growth. (a, b), Measurements of the PRA (a) and compactness (b) of plants grown in different soil types at 14 DAS. (c), Representative pictures of plants grown in potting soil, poor soil, enriched soil and propagation soil. Because the substrates had different initial SWH levels and the required water was calculated for a whole plate, an entire PhenoWell® plate was used per soil type. This resulted in measurements of more than 21 plants per soil type that were irrigated daily.

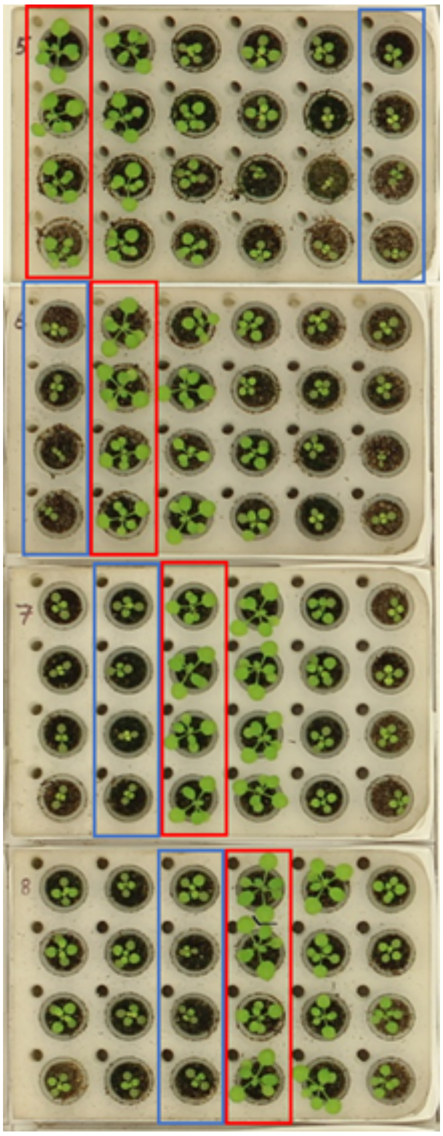

Figure S2. Example of a four-plate PhenoWell® experiment. NAA treatment in the PhenoWell® system with 0.01, 0.05, 0.1, 1 and 10 µM NAA. The red rectangles indicate the control treatment and the blue rectangle the 10-µM NAA treatment. The treatments are moved one column to the right on the following plate to avoid a possible positioning effect.

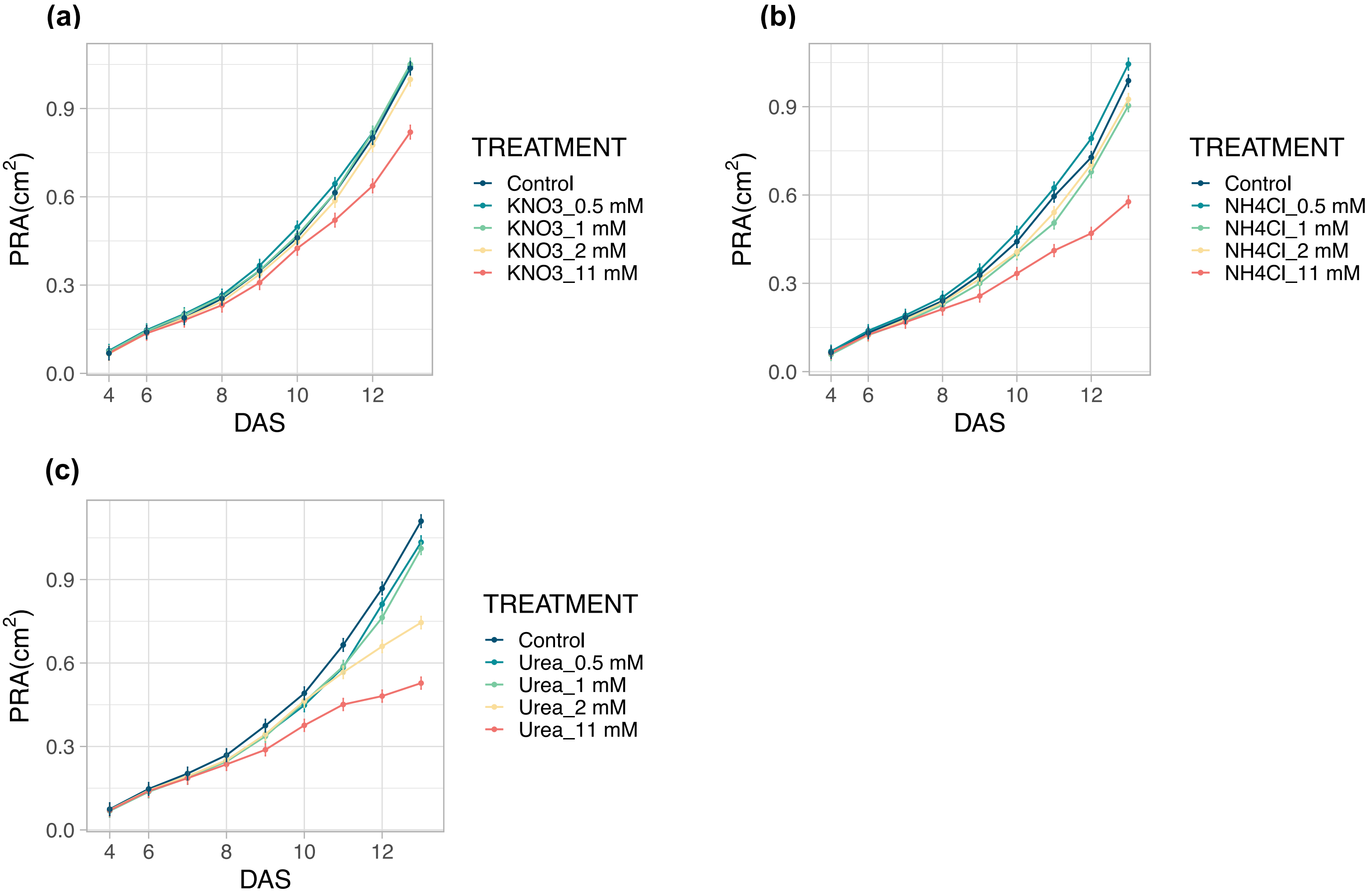

Figure S3. Effect of different nitrogen fertilizers on rosette growth. Changes in projected rosette area (PRA) of plants grown in propagation soil and treated with different concentrations of (a) KNO_3, (b) NH_4Cl and (c) urea (CH_4N_2O). Error bars indicate standard error of the mean (n =4).

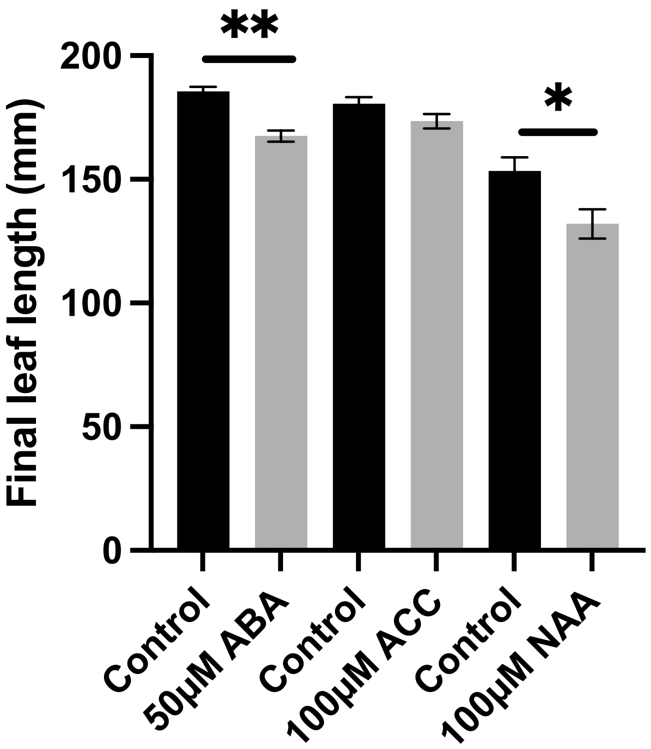

Figure S4. Changes in final leaf2 length of maize B104 after the treatment with different phytohormones and PBZ compared to the control treatment in the PhenoWell® system. Error bars indicate standard error of the mean; * and ** stands for $p < 0.05$ and 0.01, respectively (approximate F-test; n =2).

(a)

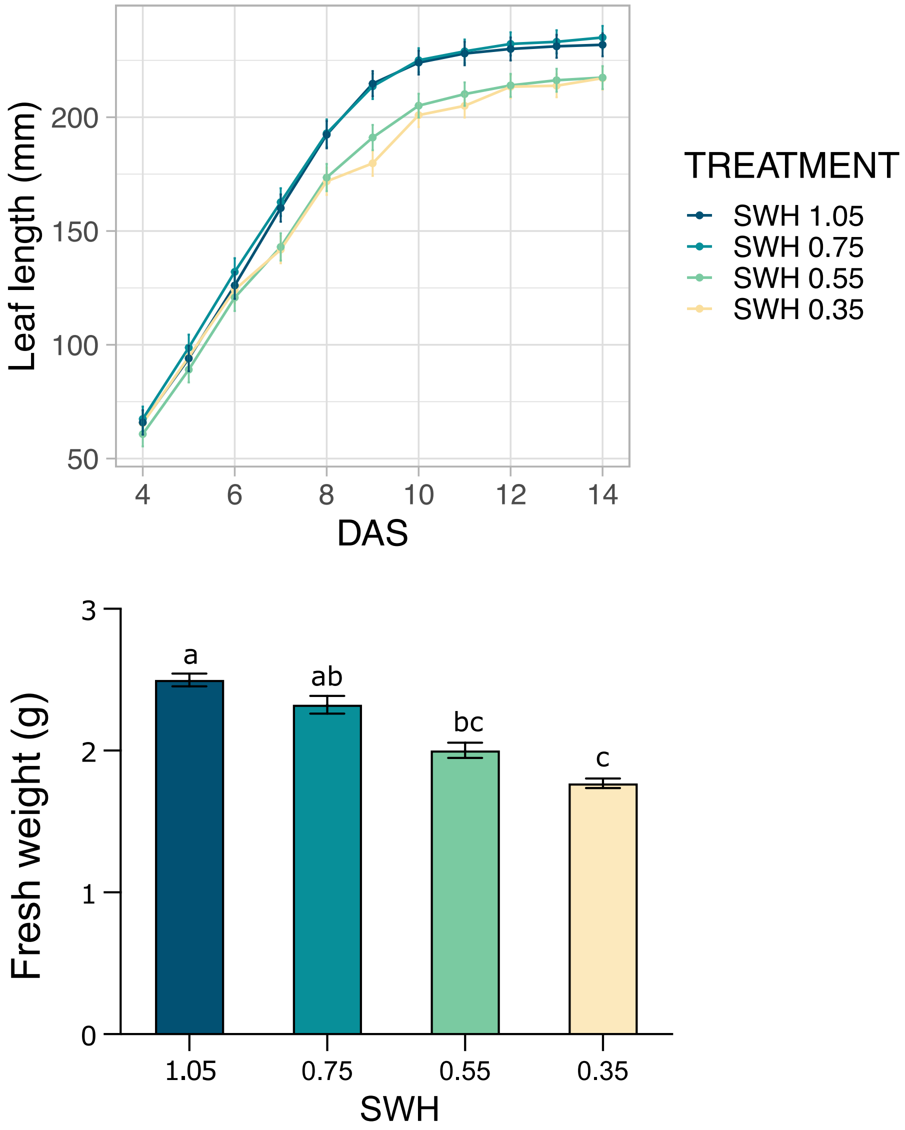

Figure S5. The effect of different drought stress regimes on plant growth in the modified maize Phenowell® system. Final leaf2 length (a) and fresh weight (b) of maize B104 plants with the different SWH levels. Error bars indicate standard error of the mean; Data sets with no statistical difference are labeled with identical letters (n =3).

Table S1. Nutrient analysis for the different soil types.

Nutrient [mg/L]	Poor Soil	Propagation Soil	Potting Soil	Enriched Soil
NO_3-N	8.7	< 5.0	32.4	369.2
NH_4-N	8.9	< 5.0	< 5.0	11.9
P	< 14.0	26.3	53.7	75.3
K	< 36.0	128.1	241.9	371.2

Table S2 Concentrations of solutions for Arabidopsis treatments

Treatment	Solute	Concentrations	Unit
phytohormone	Abscisic acid (ABA)	1, 2.5, 5, 10, 20	μM
phytohormone	1-Aminocyclo-propane-1-carboxylic acid (ACC)	1, 10, 20, 35, 50	μM
phytohormone	6-Benzylaminopurine (BAP)	0.1, 1, 2.5, 5, 10	μM
phytohormone	Brassinolide (BL)	0.1, 0.25, 0.5, 0.75, 1	μM
phytohormone	Gibberellic acid 3 (GA)	1, 25, 50, 75, 100	μM
phytohormone	1-Naphthaleneacetic acid (NAA)	0.01, 0.05, 0.1, 1, 10	μM
osmotic stress	Mannitol	5, 10, 25	mM
osmotic stress	Sorbitol	5, 10 ,25	mM
salinity	NaCl	10, 25, 35, 50, 100	mM
nitrogen fertilizer	KNO_3	0.5, 1, 2, 11	mM
nitrogen fertilizer	NH_4Cl	0.5, 1, 2, 11	mM
nitrogen fertilizer	Urea (CH_4N_2O)	0.5, 1, 2, 11	mM
Phosphate	KH_2PO_4	4, 8, 16, 32, 64	$mg \cdot L^{-1}$
